# Supplementary material for: Diversity of an uncommon elastic hypersaline microbial mat along a small-scale transect
Source: PeerJ. 2022 Jun 20;10:e13579. doi: 10.7717/peerj.13579 (PMC9220918; doi:10.7717/peerj.13579)
Supplement: Supplemental Information 1 — (A) Phylum composition within 16S rDNA gene data. [file peerj-10-13579-s001.pdf]

A

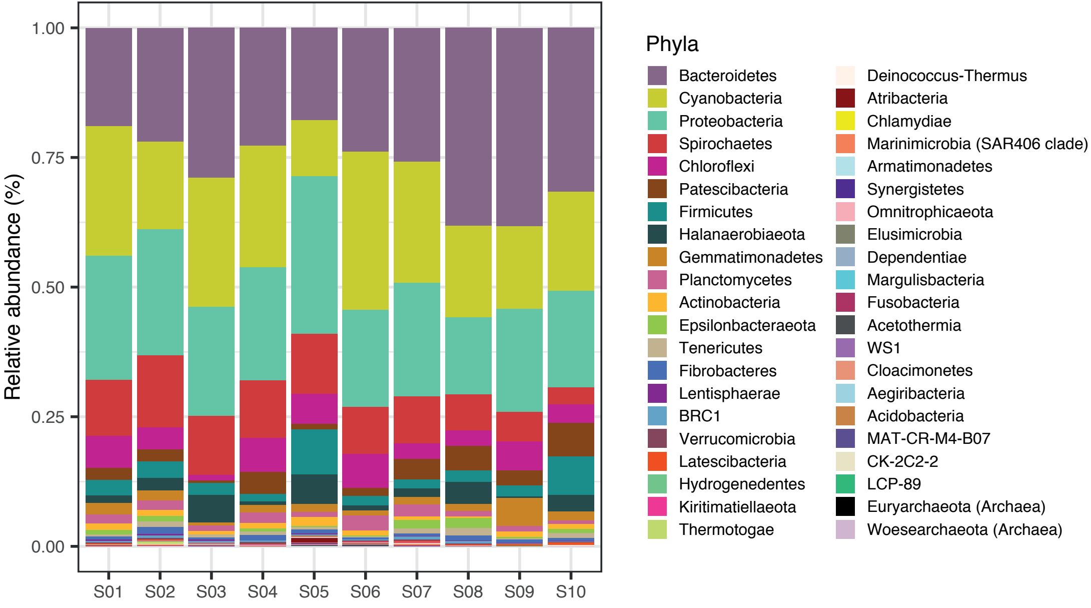

Relative abundance (%)
1.00
0.75
0.50
0.25
0.00
S01
S02
S03
S04
S05
S06
S07
S08
S09
S10
Phyla
Bacteroidetes
Cyanobacteria
Proteobacteria
Spirochaetes
Chloroflexi
Patescibacteria
Firmicutes
Halanaerobiaeota
Gemmatimonadetes
Planctomycetes
Actinobacteria
Epsilonbacteraeota
Tenericutes
Fibrobacteres
Lentisphaerae
BRC1
Verrucomicrobia
Latescibacteria
Hydrogenedentes
Kiritimatiellaeota
Thermotogae
Deinococcus-Thermus
Atribacteria
Chlamydiae
Marinimicrobia (SAR406 clade)
Armatimonadetes
Synergistetes
Omnitrophicaeota
Elusimicrobia
Dependentiae
Margulisbacteria
Fusobacteria
Acetothermia
WS1
Cloacimonetes
Aegiribacteria
Acidobacteria
MAT-CR-M4-B07
CK-2C2-2
LCP-89
Euryarchaeota (Archaea)
Woesearchaeota (Archaea)
