# Supplementary figures and images for: Diversity of an uncommon elastic hypersaline microbial mat along a small-scale transect
Source: PeerJ. 2022 Jun 20;10:e13579. doi: 10.7717/peerj.13579 (PMC9220918; doi:10.7717/peerj.13579)

**B**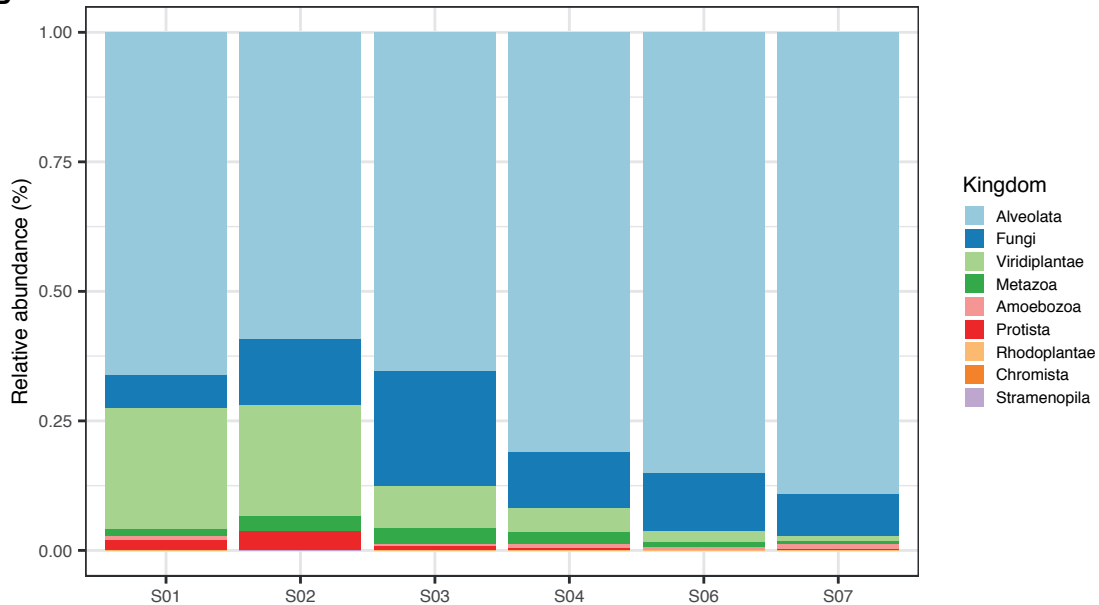

Supplement: Supplemental Information 2 — (B) Kingdom composition (based on UNITE database eukaryotic taxonomy). [file peerj-10-13579-s002.pdf]

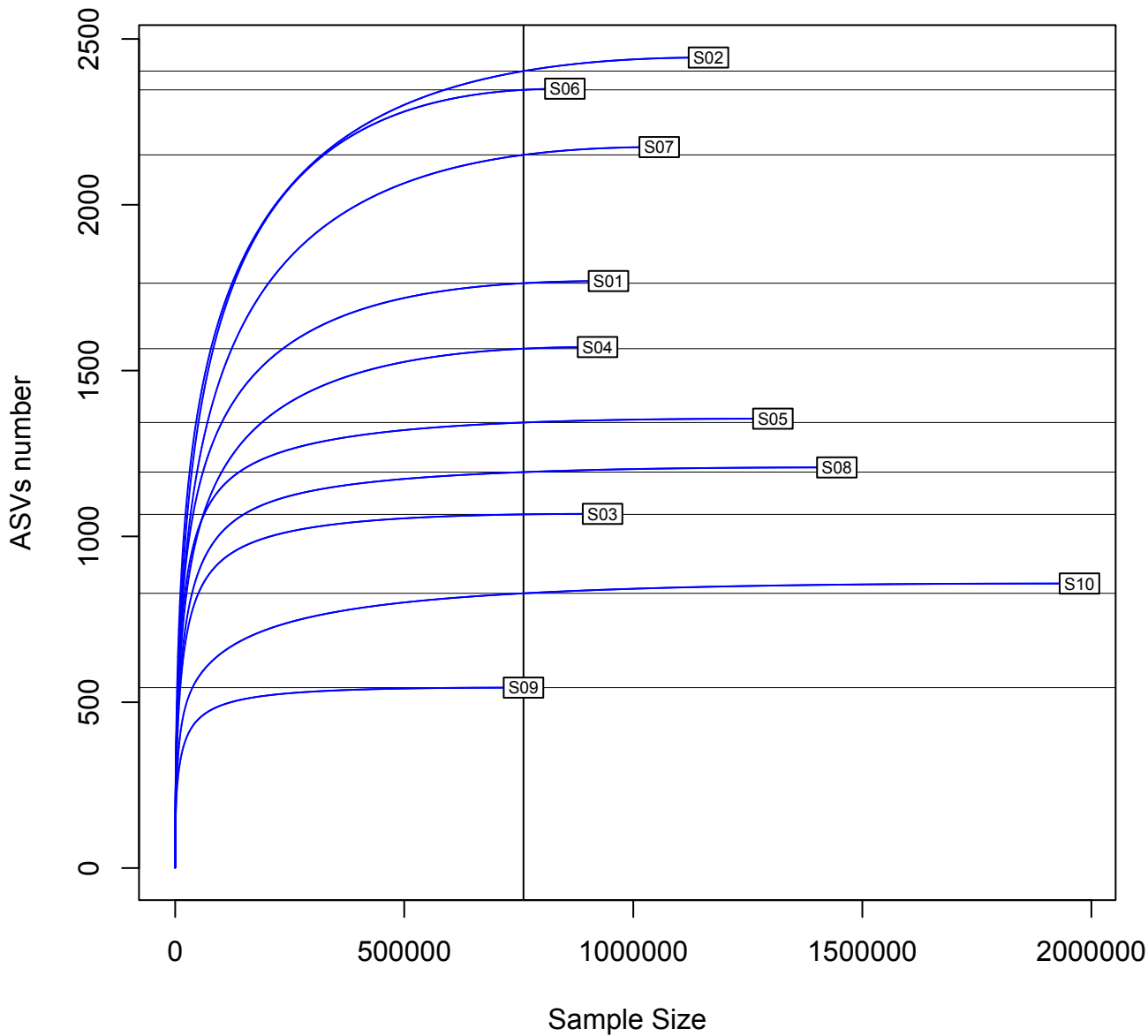

Supplement: Supplemental Information 3 [file peerj-10-13579-s003.pdf]

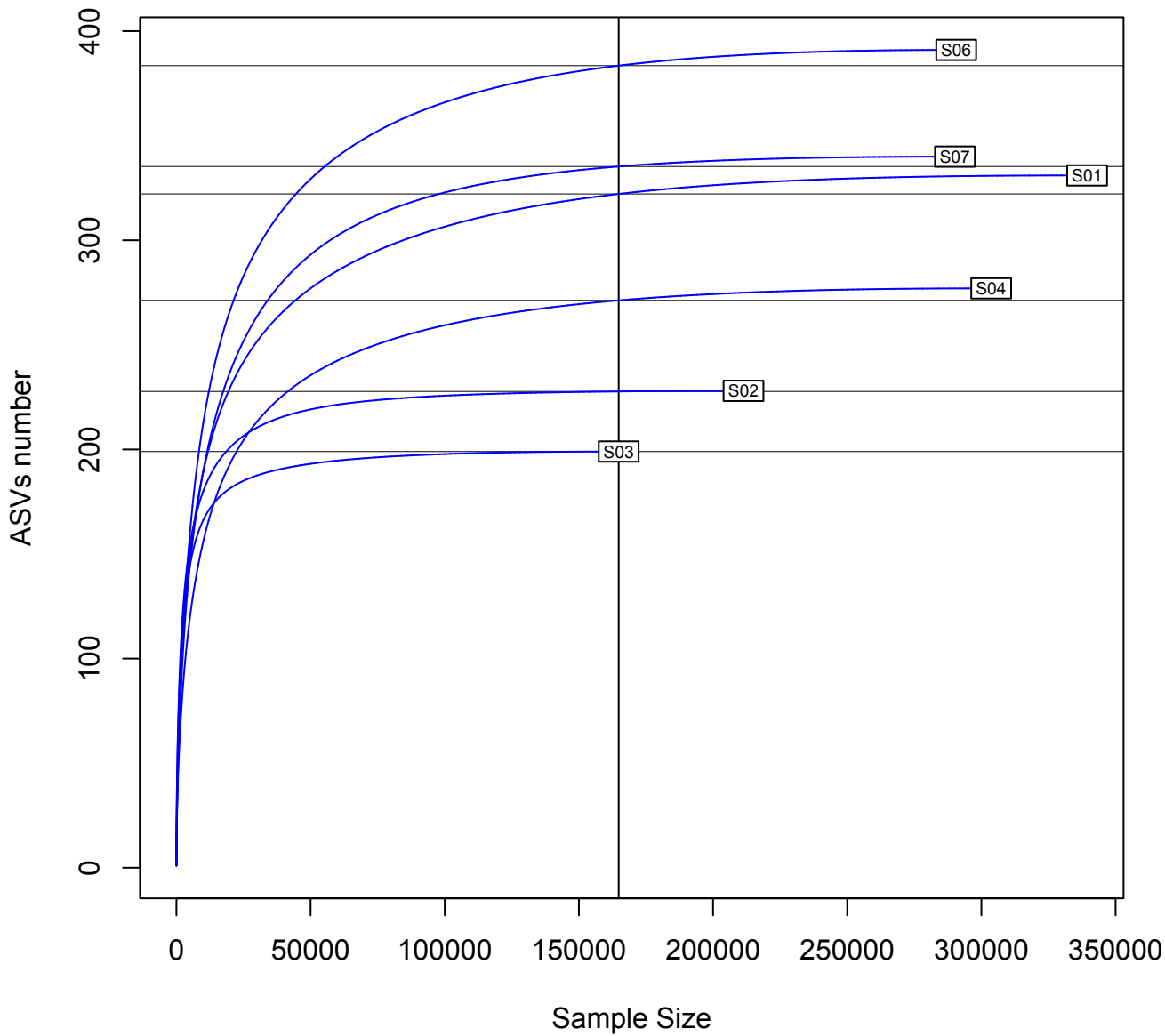

Supplement: Supplemental Information 4 [file peerj-10-13579-s004.pdf]

# UPGMA Bray

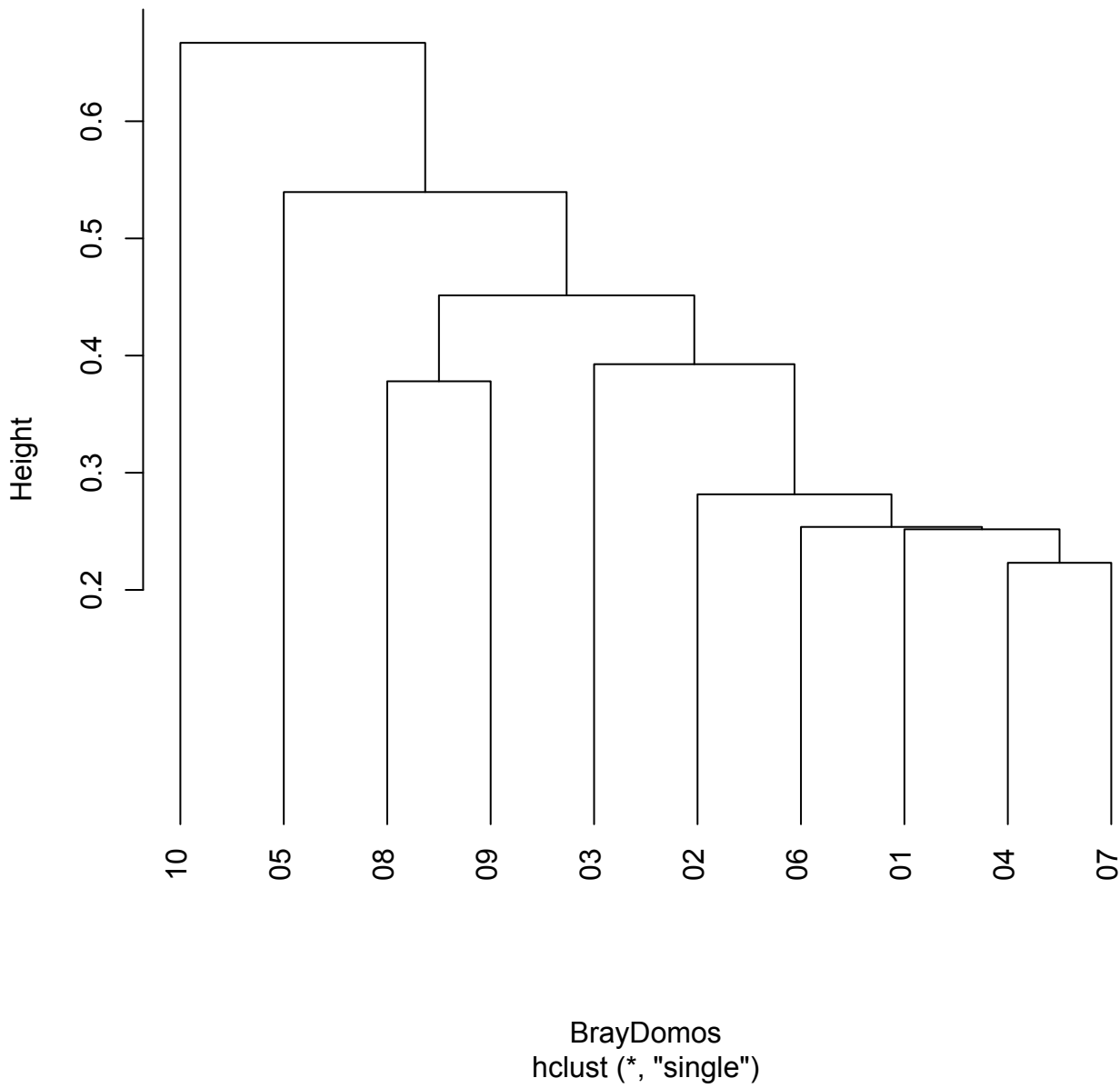

Supplement: Supplemental Information 5 — Branch length represents Bray-Curtis dissimilarity coefficient, based on 16S rDNA ASVs. [file peerj-10-13579-s005.pdf]

# UPGMA Jaccard

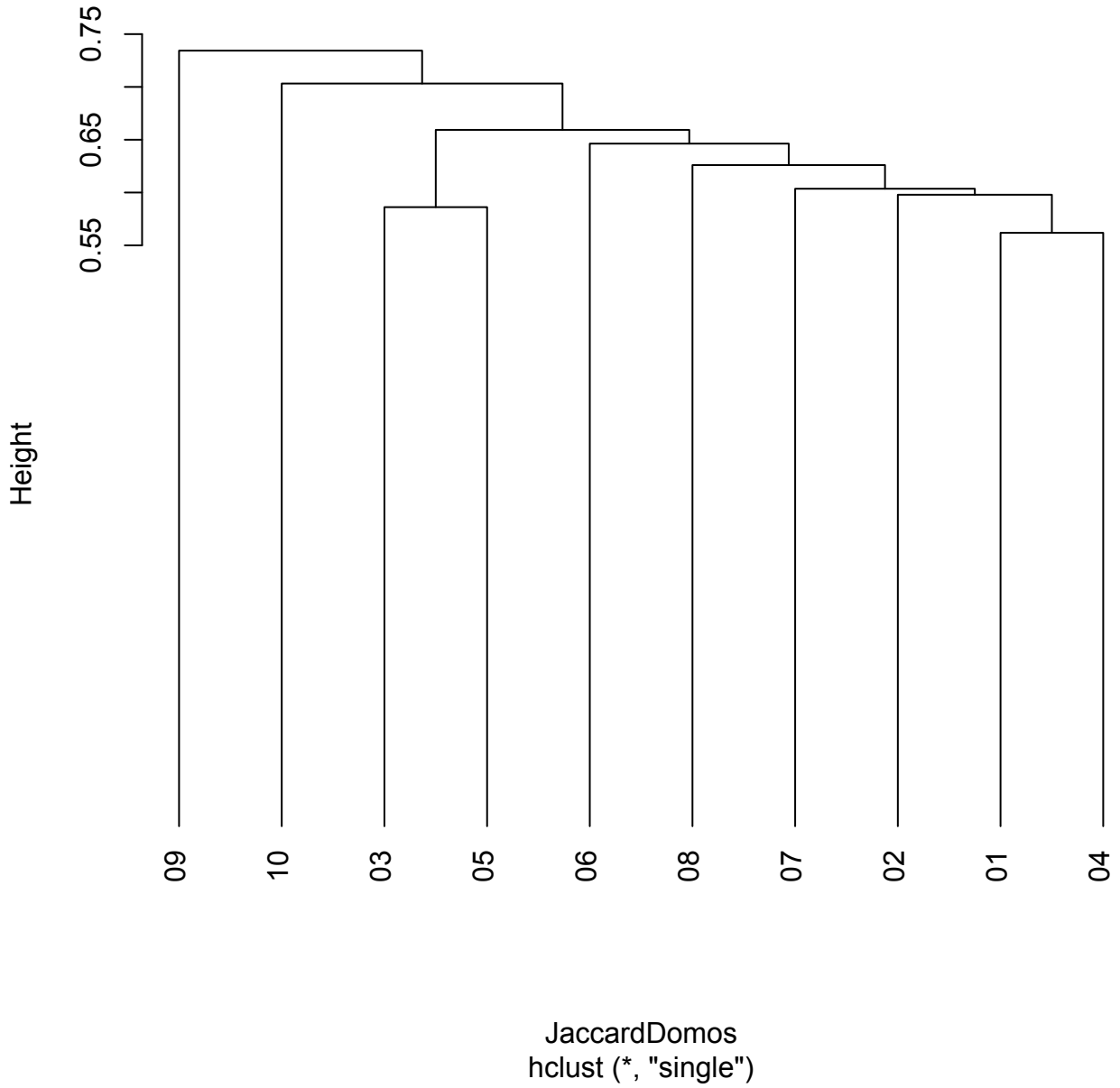

Supplement: Supplemental Information 6 — Branch length represents Jaccard-1 dissimilarity coefficient, based on 16S rDNA ASVs. [file peerj-10-13579-s006.pdf]

# UPGMA Bray Curtis

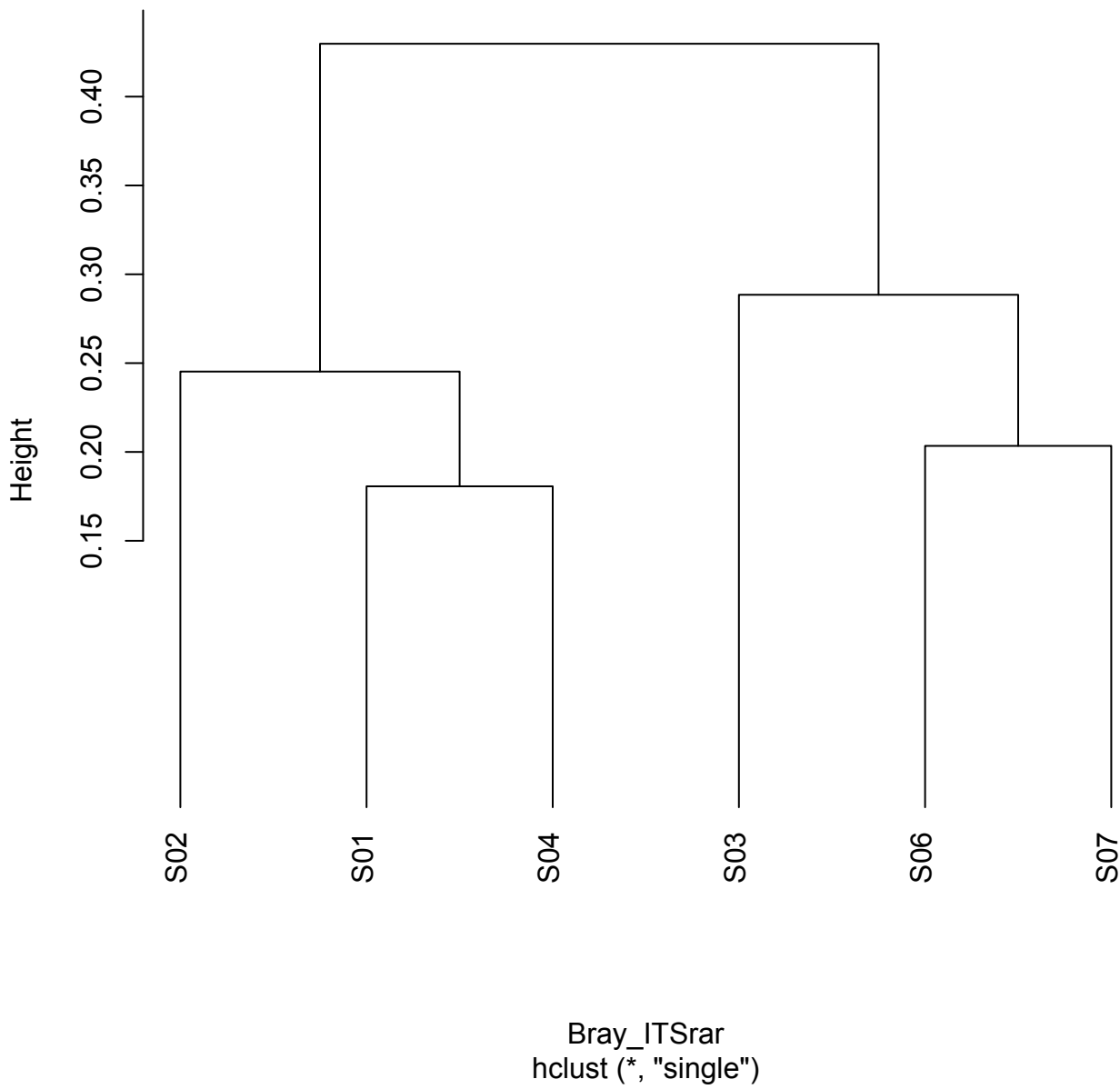

Supplement: Supplemental Information 7 — Branch length represents Bray-Curtis dissimilarity coefficient, based on Forward-Only ITS ASVs. [file peerj-10-13579-s007.pdf]

# UPGMA Jaccard

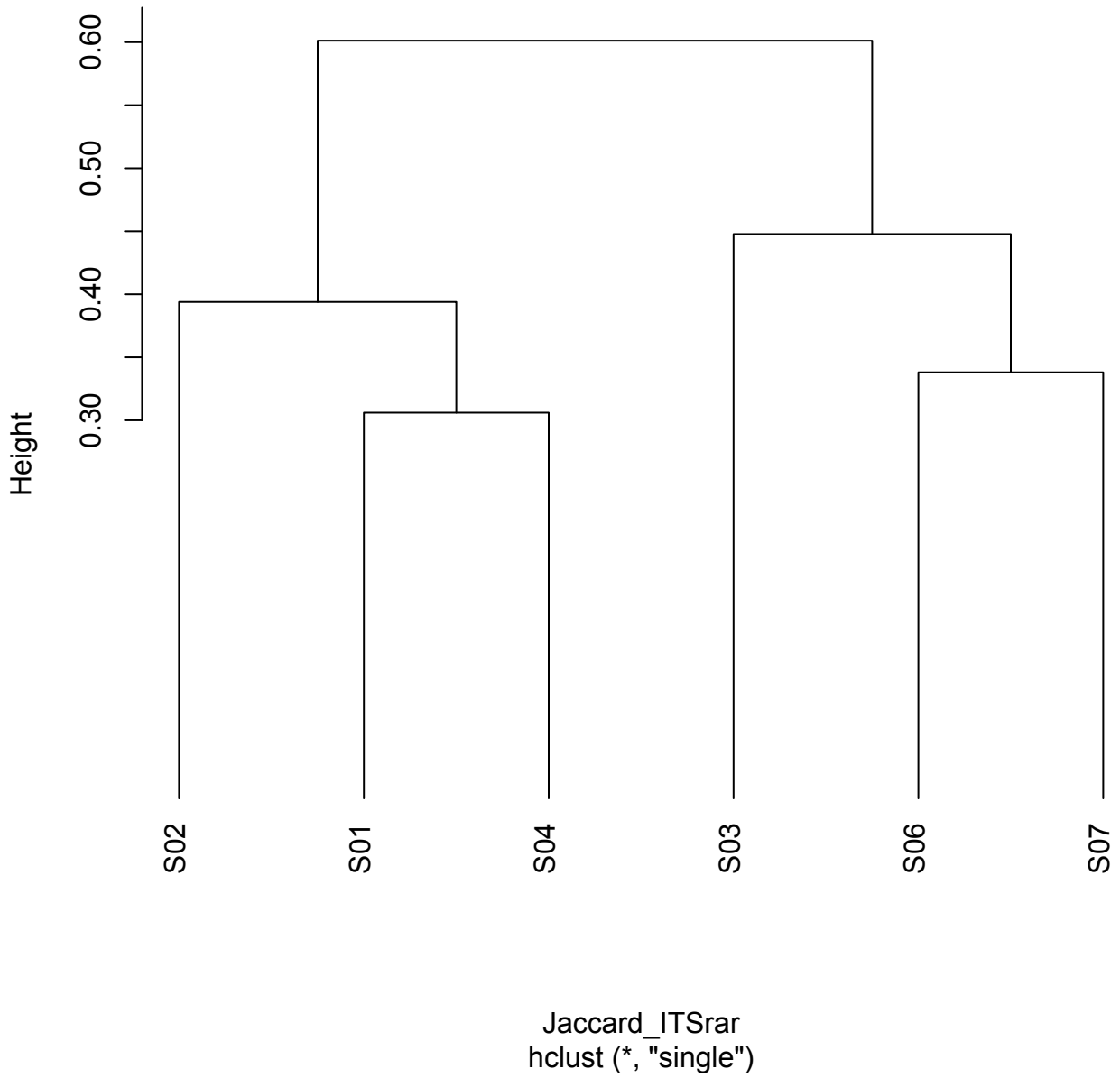

Supplement: Supplemental Information 8 — Branch length represents Jaccard-1 dissimilarity coefficient, based on Forward-Only ITS ASVs. [file peerj-10-13579-s008.pdf]

# A

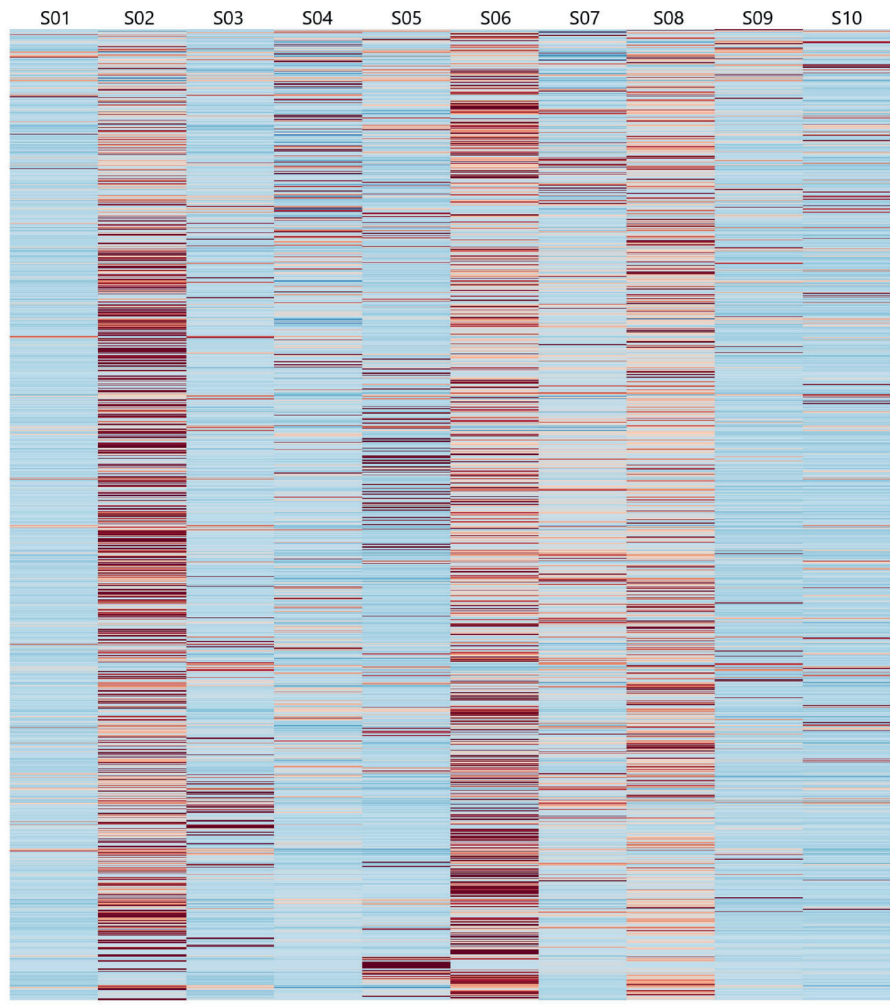

ENLISTED IONS

# B

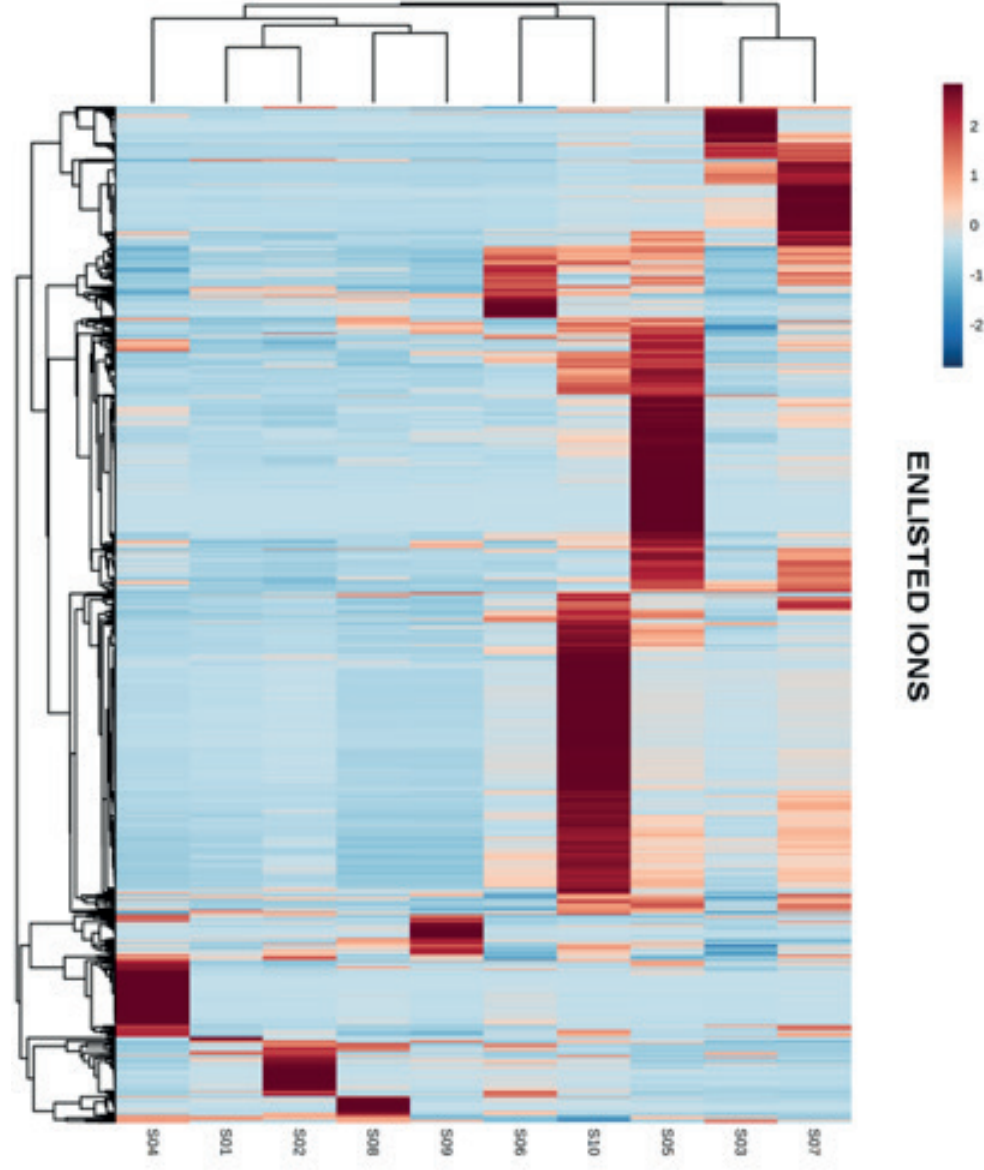

ENLISTED IONS

Supplement: Supplemental Information 9 — Relative proportion of metabolite features (MS ions in positive mode) in the Archaean Domes mats samples. On the scale bar, brick red color indicates increased metabolite levels, and blue color represents decreased levels. (A) non-clustered samples and sites, (B) clustered samples and sites. [file peerj-10-13579-s009.pdf]
