# Supplementary material for: Diversity of an uncommon elastic hypersaline microbial mat along a small-scale transect
Source: PeerJ. 2022 Jun 20;10:e13579. doi: 10.7717/peerj.13579 (PMC9220918; doi:10.7717/peerj.13579)
Supplement: Supplemental Information 12 — 16S rDNA gene phyla and ITS region kingdom taxa community composition ASVs tables, and percentage of classified and unclassified ASVs. [file peerj-10-13579-s012.pdf]

ITS region kingdom amplicon abundance and ASV number (Forward only database, using UNITE database Eukaryal taxonomy)

	S01	S02	S03	S04	S06	S07
Alveolata	73308	53833	71131	84579	155785	194989
Amoebozoa	938	139	433	776	874	2342
Protista	2203	3215	929	458	237	491
Chromista	0	44	0	0	0	0
Viridiplantae	25899	19608	8959	5098	4150	2441
Rhodoplantae	18	9	26	3	11	22
Stramenopila	0	6	0	0	0	0
Fungi	6938	11471	24026	11173	20383	17496
Metazoa	1347	2507	3147	2348	1667	965
TOTAL Classified	110651	90832	108651	104435	183107	218746
Unclassified	242069	127246	59009	200803	107999	71296
TOTAL	352720	218078	167660	305238	291106	290042

816422	Sum of classified reads
1624844	Sum of all reads (ASVs assigned)
50.25%	Percentage classified reads

ASV number		
Alveolata	137	Protozoa ASVs = 329
Amoebozoa	28	
Protista	22	
Chromista	1	
Rhodoplantae	4	
Stramenopila	137	
Viridiplantae	1	
Fungi	109	
Metazoa	23	
NA	461	
TOTAL ASV number	923	

16S rRNA gene phylum amplicon abundance (rarified). Red cells indicate phylum that contains abundant (<1% overall) ASVs

		1	2	3	4	5	6	7	8	9	10
ARCHAEA											
1	D_0__Archaea;__	64	60	0	17	11	6	29	8	0	0
2	D_0__Archaea;D_1__Euryarchaeota	3	41	0	0	26	97	0	0	0	0
3	D_0__Archaea;D_1__Woesearchaeota	8	24	0	27	0	16	60	9	0	6
BACTERIA											
1	D_0__Bacteria;D_1__Acetothermia	129	10	52	15	1144	2277	7	0	0	0
2	D_0__Bacteria;D_1__Acidobacteria	0	0	0	3	0	0	0	10	31	0
3	D_0__Bacteria;D_1__Actinobacteria	8407	7669	4096	6915	12210	6621	4414	1849	7331	3030
4	D_0__Bacteria;D_1__Aegiribacteria	142	38	0	0	684	10	0	0	0	0
5	D_0__Bacteria;D_1__Armatimonadetes	135	99	120	294	43	420	365	26	126	211
6	D_0__Bacteria;D_1__Atribacteria	572	153	245	273	7077	1322	135	115	13	19
7	D_0__Bacteria;D_1__Bacteroidetes	134668	153556	191232	163899	125917	165242	180444	281383	283266	101350
8	D_0__Bacteria;D_1__BRC1	1777	3285	2086	1602	1153	1081	3394	3392	231	216
9	D_0__Bacteria;D_1__Chlamydiae	281	301	24	122	36	192	379	256	32	0
10	D_0__Bacteria;D_1__Chloroflexi	43304	29063	6413	47211	41227	44979	20777	22024	41194	11244
11	D_0__Bacteria;D_1__CK-2C2-2	0	5	0	0	0	0	0	0	0	0
12	D_0__Bacteria;D_1__Cloacimonetes	112	658	58	8	0	0	4	0	0	0
13	D_0__Bacteria;D_1__Cyanobacteria	178468	118933	164747	169076	77423	211897	164081	129929	118366	61562
14	D_0__Bacteria;D_1__Deinococcus-Thermus	363	496	141	167	277	304	128	218	0	336
15	D_0__Bacteria;D_1__Dependentiae	13	0	32	62	142	39	96	40	0	0
16	D_0__Bacteria;D_1__Elusimicrobia	32	38	43	45	754	68	34	89	0	0
17	D_0__Bacteria;D_1__Epsilonbacteraeota	6384	7925	248	5249	1210	1218	11976	14629	2773	2836
18	D_0__Bacteria;D_1__Fibrobacteres	4744	9465	2546	7410	5276	2741	3378	6510	4530	1803
19	D_0__Bacteria;D_1__Firmicutes	21350	22247	15217	9825	61093	12689	11840	15965	15169	23660
20	D_0__Bacteria;D_1__Fusobacteria	57	33	13	16	20	4	37	23	0	0
21	D_0__Bacteria;D_1__Gemmatimonadetes	15451	13014	3766	10268	10624	6286	9812	9923	39397	5622
22	D_0__Bacteria;D_1__Halanaerobiaeota	10500	17191	35438	5328	40211	6557	11496	30810	2480	10343
23	D_0__Bacteria;D_1__Hydrogenedentes	351	805	460	400	1076	325	326	226	13	136
24	D_0__Bacteria;D_1__Kiritimatiellaeota	383	553	271	604	229	315	920	273	17	0
25	D_0__Bacteria;D_1__Latescibacteria	755	1052	219	493	570	557	799	1215	3280	1582
26	D_0__Bacteria;D_1__LCP-89	0	0	0	0	0	0	0	6	12	0
27	D_0__Bacteria;D_1__Lentisphaerae	1972	2306	2357	495	1426	327	1284	1457	1462	257
28	D_0__Bacteria;D_1__Margulisbacteria	166	0	17	26	74	23	100	41	0	3
29	D_0__Bacteria;D_1__Marinimicrobia (SAR406 cla	97	906	431	173	283	75	1657	26	0	0
30	D_0__Bacteria;D_1__MAT-CR-M4-B07	0	70	8	14	0	0	0	0	0	0
31	D_0__Bacteria;D_1__Omnitrophicaeota	30	37	74	47	188	23	277	65	0	43
32	D_0__Bacteria;D_1__Patescibacteria	17159	16264	3493	31165	7895	11468	28058	35446	22413	20970
33	D_0__Bacteria;D_1__Planctomycetes	12906	13262	7265	14788	7614	20303	16854	8469	8828	2296
34	D_0__Bacteria;D_1__Proteobacteria	169708	169915	138870	157279	215777	129417	152922	109274	147175	59164
35	D_0__Bacteria;D_1__Spirochaetes	77444	98384	75407	79671	82623	63525	63786	51066	42598	10856
36	D_0__Bacteria;D_1__Synergistetes	169	350	921	42	1483	179	80	0	0	0
37	D_0__Bacteria;D_1__Tenericutes	2320	7003	3500	4200	3312	1124	6143	10586	787	3028
38	D_0__Bacteria;D_1__Thermotogae	198	3317	703	455	896	133	1527	82	0	0
39	D_0__Bacteria;D_1__Verrucomicrobia	1524	2645	614	3222	468	1713	2555	1140	125	340
40	D_0__Bacteria;D_1__WS1	19	19	0	9	0	21	9	0	0	4
UNCLASSIFIED											
1	Unassigned;__	4980	6157	996	4086	227	9761	7874	959	1773	2587
2	D_0__Bacteria;__	42855	52651	97877	34999	49301	56645	51943	22461	16578	436496
TOTAL UNCLASSIFIED		47899	58868	98873	39102	49539	66412	59846	23428	18351	439083
PERCENTAGE UNCLASSIFIED WITHIN 760000		6.3025	7.7457895	13.009605	5.145	6.5182895	8.7384211	7.8744737	3.08263158	2.414605263	57.77407895
	READS PER SITE (NON RARIFIED)	945825	1164094	932904	921970	1304754	850608	1056618	1444352	760326	1973982

READS ABUNDANCE		ASVs NUMBER
901401	Non classified	4132
7600000	Sum of all	6063
11.86053947	Percentage non classified	68.15
