# Supplementary material for: Diversity of an uncommon elastic hypersaline microbial mat along a small-scale transect
Source: PeerJ. 2022 Jun 20;10:e13579. doi: 10.7717/peerj.13579 (PMC9220918; doi:10.7717/peerj.13579)
Supplement: Supplemental Information 15 [file peerj-10-13579-s015.pdf]

Non-significatives Mantel test results for geographic distance vs beta diversity

16S Bray p=0.0724   r= 0.2749
ITS Jaccard p= 0.13056   r= 0.3621
ITS Bray p= 0.13194   r=0.3623

Non-significatives Mantel test results for metabolomic data vs beta diversity

16S Jaccard p= 0.6126 r= -0.08238005
16S Bray p= 0.4274 r= 0.02121974
ITS Jaccard p=0.3611 r=0.0794
ITS Bray p=0.303 r=0.1178
