# Supplementary material for: Diversity of an uncommon elastic hypersaline microbial mat along a small-scale transect
Source: PeerJ. 2022 Jun 20;10:e13579. doi: 10.7717/peerj.13579 (PMC9220918; doi:10.7717/peerj.13579)
Supplement: Supplemental Information 16 [file peerj-10-13579-s016.pdf]

	psc_16S	psc_rar_16S	psc_abun_16	psc_ITS	psc_rar_ITS	psc_abun_ITS
S1	0.11060947	0.30236487	0.04866875	0.10968344	0.30487551	0.10002044
S2	0.10863735	0.29149669	0.0605547	0.09490329	0.2868876	0.10927695
S3	0.09063427	0.27552425	0.05493335	0.08496886	0.26553793	0.12463795
S4	0.11408563	0.32433312	0.05225602	0.11582347	0.30695845	0.09225003
S5	0.07809136	0.24593114	0.04262518			
S6	0.11513793	0.30216016	0.08670753	0.11553622	0.29763445	0.1295801
S7	0.11590233	0.30581557	0.07509745	0.12709692	0.37551424	0.11824146
S8	0.07955718	0.24829185	0.04620871			
S9	0.13062694	0.34927144	0.07490645			
S10	0.1354835	0.33490847	0.08888336			
