# Supplementary material for: Diversity of an uncommon elastic hypersaline microbial mat along a small-scale transect
Source: PeerJ. 2022 Jun 20;10:e13579. doi: 10.7717/peerj.13579 (PMC9220918; doi:10.7717/peerj.13579)
Supplement: Supplemental Information 19 — Low read count genera (less than 1% of total proportion of taxa) labels from Figure 2A (up). Five repeated sequences of colors are used, distinguished here by red lines, and numerated red arrows in figure. [file peerj-10-13579-s019.pdf]

1

- Acinetobacter (Proteobacteria)
- Aerococcus (Firmicutes)
- Albimonas (Proteobacteria)
- Aliifodinibius (Bacteroidetes)
- Alkaliflexus (Bacteroidetes)
- Allofrancisella (Proteobacteria)
- Anaerobranca (Firmicutes)
- Anaerosalibacter (Firmicutes)
- Aquicella (Proteobacteria)
- Bacillus (Firmicutes)
- bacterium YC-ZSS-LKJ186 (Aegiribacteria)
- Bdellovibrio (Proteobacteria)
- Blastopirellula (Planctomycetes)
- Brevinema (Spirochaetes)
- Brochothrix (Firmicutes)
- Candidatus (Paenicardinium (Bacteroidetes)
- Candidatus Endoecteinascidia (Proteobacteria)
- Candidatus Endomicrobium (Elusimicrobia)
- Candidatus Fritschea (Chlamydiae)
- Candidatus Megaira (Proteobacteria)
- Candidatus Melainabacteria bacterium RIFOXYA2_FULL_32_9 (Cyanobacteria)
- Candidatus Phytoplasma (Tenericutes)
- Carboxydocella (Firmicutes)
- Cerasicoccus (Verrucomicrobia)
- Chloroflexi bacterium RBG_13_46_14 (Chloroflexi)
- Clostridiales bacterium PH28_bin88 (Chloroflexi)
- Clostridiisalibacter (Firmicutes)
- Clostridium sensu stricto 7 (Firmicutes)
- Defluviitalea (Firmicutes)
- Defluviitaleaceae UCG-011 (Firmicutes)
- Dehalobium (Chloroflexi)
- Desulfocella (Proteobacteria)
- Desulfonatronovibrio (Proteobacteria)
- Desulfuribacillus (Firmicutes)
- Enterococcus (Firmicutes)
- Escherichia coli (Proteobacteria)
- Estrella (Chlamydiae)
- FFCH7168 (Chloroflexi)
- Fodinicurvata (Proteobacteria)
- Francisella (Proteobacteria)
- Garciella (Firmicutes)
- Geitlerinema PCC-7105 (Cyanobacteria)

2

- Halarsenatibacter (Halanaerobiaeota)
- Haliea (Proteobacteria)
- Halonatronum (Halanaerobiaeota)
- Holospora (Proteobacteria)
- Hydrogenispora (Firmicutes)
- Lachnospiraceae NK3A20 group (Firmicutes)
- Legionella (Proteobacteria)
- Leptococcus JA-3-3Ab (Cyanobacteria)
- Leuconostoc (Firmicutes)
- Lewinella (Bacteroidetes)
- Litoricola (Proteobacteria)
- Lutispora (Firmicutes)
- MAT-CR-P4-C12 (Bacteroidetes)
- Melioribacter (Bacteroidetes)
- Modestobacter (Actinobacteria)
- MSBL3 (Kiritimatiellaeota)
- Neochlamydia (Chlamydiae)
- Nitrolancea (Chloroflexi)
- Ohtaekwangia (Bacteroidetes)
- Oscillatoria PCC-6304 (Cyanobacteria)
- Paenibacillus (Firmicutes)
- Parcubacteria group bacterium GW2011_GWF2_45_11 (Patescibacteria)
- Phaselicystis (Proteobacteria)
- Planktosalinus (Bacteroidetes)
- Prevotella 1 (Bacteroidetes)
- Prevotella 7 (Bacteroidetes)
- Prevotellaceae UCG-003 (Bacteroidetes)
- Pseudobacteriovorax (Proteobacteria)
- Pseudobacteroides (Firmicutes)
- Pseudohongiella (Proteobacteria)
- Pseudomonas (Proteobacteria)
- Psychrobacter (Proteobacteria)
- Psychroflexus (Bacteroidetes)
- Rhodococcus (Actinobacteria)
- Rhodopirellula (Planctomycetes)
- Rikenellaceae RC9 gut group (Bacteroidetes)
- Roseiflexus (Chloroflexi)
- Roseimarinus (Bacteroidetes)
- Roseobacter clade CHAB-I-5 lineage (Proteobacteria)
- Rubellimicrobium (Proteobacteria)
- Rubrobacter (Actinobacteria)
- Ruminiclostridium 1 (Firmicutes)

3

- Ruminococcaceae NK4A214 group (Firmicutes)
- Ruminococcaceae UCG-013 (Firmicutes)
- Ruminococcaceae UCG-014 (Firmicutes)
- Salinisphaera (Proteobacteria)
- Salipaludibacillus (Firmicutes)
- Salisediminibacterium (Firmicutes)
- SEEP-SRB1 (Proteobacteria)
- Silvanigrella (Proteobacteria)
- ST-3K26 (Bacteroidetes)
- Streptomyces (Actinobacteria)
- Sulfurimonas (Epsilonbacteraeota)
- Taeseokella (Bacteroidetes)
- Thalassospira (Proteobacteria)
- Thermovirga (Synergistetes)
- Thermus (Deinococcus-Thermus)
- Tistrella (Proteobacteria)
- Veillonella (Firmicutes)
- Wenzhouxiangella (Proteobacteria)
- Wohlfahrtiimonas (Proteobacteria)
- Geotoga (Thermotogae)
- bacterium enrichment culture clone E23 (Cloacimonetes)
- Marinococcus (Firmicutes)
- Acholeplasma (Tenericutes)
- Salinispira (Spirochaetes)
- Salinivibrio (Proteobacteria)
- Chitinivibrio (Fibrobacteres)
- F1-37X2 (Bacteroidetes)
- Pontibacter (Bacteroidetes)
- ESFC-1 (Cyanobacteria)
- Fictibacillus (Firmicutes)
- Anaerolinea (Chloroflexi)
- Fusibacter (Firmicutes)
- Caenispirillum (Proteobacteria)
- Sodalis (Proteobacteria)
- Halofilum (Proteobacteria)
- Desulfofustis (Proteobacteria)
- Puniceicoccus (Verrucomicrobia)
- Alkaliphilus (Firmicutes)
- Rubrivirga (Bacteroidetes)
- Candidatus Melainabacteria bacterium GWA2_34_9 (Cyanobacteria)
- Desulfotomaculum (Firmicutes)
- Oscillochloris (Chloroflexi)

4

- Halochromatium (Proteobacteria)
- Thiohalorhabdus (Proteobacteria)
- Cesiribacter (Bacteroidetes)
- Izimaplasma (Tenericutes)
- IheB3-7 (Bacteroidetes)
- Brachyspira (Spirochaetes)
- Pseudokineococcus (Actinobacteria)
- bacterium YC-ZSS-LKJ145 (Chloroflexi)
- SBZC-1223 (Lentisphaerae)
- Peredibacter (Proteobacteria)
- Desulfonauticus (Proteobacteria)
- Marinobacter (Proteobacteria)
- Dehalobacter (Firmicutes)
- GWE2-31-10 (Spirochaetes)
- Marivita (Proteobacteria)
- Ruminiclostridium (Firmicutes)
- Chloronema (Chloroflexi)
- Candidatus Alysiosphaera (Proteobacteria)
- Desulfotignum (Proteobacteria)
- Longimonas (Bacteroidetes)
- Phaeodactylibacter (Bacteroidetes)
- Methylobacterium (Proteobacteria)
- Candidatus Latescibacter (Latescibacteria)
- Salinibacter (Bacteroidetes)
- Owenweeksia (Bacteroidetes)
- Phormidium MBIC10003 (Cyanobacteria)
- Halopeptonella (Proteobacteria)
- Natronincola (Firmicutes)
- Desulfatiglans (Proteobacteria)
- Parvularcula (Proteobacteria)
- Rapidithrix (Bacteroidetes)
- Deltaproteobacteria bacterium RIFOXYB12_FULL_58_9 (Proteobacteria)
- Pelagicoccus (Verrucomicrobia)
- Microgenomates group bacterium RIFCSPLOWO2_01_FULL_47_10 (Patescibacteria)
- Desulfonatronum (Proteobacteria)
- Lentimonas (Verrucomicrobia)
- Orenia (Halanaerobiaeota)
- Thiomicrospira (Proteobacteria)
- Alterococcus (Verrucomicrobia)
- Roseibaca (Proteobacteria)
- Caminicella (Firmicutes)
- Dethiosulfatibacter (Firmicutes)

5

- SM1A02 (Planctomycetes)
- Natranaerobius (Firmicutes)
- Halorhodospira (Proteobacteria)
- Peptoniphilus (Firmicutes)
- Halocella (Halanaerobiaeota)
- Desulfosalsimonas (Proteobacteria)
- Truepera (Deinococcus-Thermus)
- Dethiobacter (Firmicutes)
- Ruminococcaceae UCG-012 (Firmicutes)
- Haloplasma (Tenericutes)
- SC103 (Thermotogae)
- Planctomycetales bacterium Ellin7244 (Planctomycetes)
- Halothiobacillus (Proteobacteria)
- Gracilimonas (Bacteroidetes)
- [Cytophaga] xylanolytica group (Bacteroidetes)
- Alkalibacterium (Firmicutes)
- Rhodovulum (Proteobacteria)
- Cryomorpha (Bacteroidetes)
- Candidatus (Amoebophilus (Bacteroidetes)
- Verruc-01 (Verrucomicrobia)
- Urania-1B-19 marine sediment group (Planctomycetes)
- Rubribacterium (Proteobacteria)
- Algoriphagus (Bacteroidetes)
- Desulfitibacter (Firmicutes)
- bacterium YC-ZSS-LKJ74 (Chloroflexi)
- Roseivivax (Proteobacteria)
- CENA518 (Cyanobacteria)
- Sandaracinus (Proteobacteria)
- Flavobacterium (Bacteroidetes)
- ADurb.Bin120 (Chloroflexi)
- OM27 clade (Proteobacteria)
- Candidatus Chlorothrix (Chloroflexi)
- Desulfovibrio (Proteobacteria)
- Algisphaera (Planctomycetes)
- Halomonas (Proteobacteria)
- ML310M-34 (Bacteroidetes)
- Oceanicaulis (Proteobacteria)
- Phycisphaera (Planctomycetes)
- Blvii28 wastewater-sludge group (Bacteroidetes)
- Rhodohalobacter (Bacteroidetes)
- Dactylococcopsis PCC-8305 (Cyanobacteria)
- Alkalispirochaeta (Spirochaetes)
